# Improvement of Postharvest Quality of Plum (*Prunus domestica* L.) Using Polysaccharide-Based Edible Coatings

**DOI:** 10.3390/plants9091148

**Published:** 2020-09-04

**Authors:** Sima Panahirad, Rahim Naghshiband-Hassani, Sara Bergin, Ramesh Katam, Nasser Mahna

**Affiliations:** 1 Department of Horticultural Sciences, Faculty of Agriculture, University of Tabriz, Tabriz 5166616471, Iran; sima.panahirad@gmail.com (S.P.); rahnaghsh@yahoo.com (R.N.-H.); 2 Department of Biological Sciences, Florida A&M University, Tallahassee, FL 32307, USA; sara.bergin@gmail.com

**Keywords:** carboxymethylcellulose, pectin, plum, qualitative attributes, enzymatic activity, postharvest

## Abstract

Polysaccharide-based edible coatings are served as an attractive preservation method for postharvest maintenance of most fruits. The current study examined the effect of carboxymethylcellulose (CMC)- and pectin (Pec)-based edible coatings on titratable acidity (TA), firmness; vitamin C (vit C); total soluble solids (TSS); pH; total phenolics; anthocyanin and flavonoid contents; total antioxidant capacity (based on 1,1-Diphenyl-2-picryl-hydrazyl hydrate (DPPH)); the activities of peroxidase (POD), polyphenol oxidase (PPO) and polygalacturonase (PG) enzymes; and weight loss during cold storage. The results showed that each coating and their combinations caused positive effects in all measured parameters except weight loss. The applied coatings preserved firmness and improved total phenols, anthocyanin and flavonoid contents, antioxidant capacity and POD activity. In addition, TSS decreased and pH values remained more or less stable with the coating application. The coatings delayed TA and vitamin C loss, and decreased enzymatic activities such as PPO and PG. It could be stated that CMC at 1% and Pec at 1.5% separately demonstrated the best results for most of the measured parameters; and 0.5% Pec + 1.5% CMC could be considered as the best combination. In conclusion, application of CMC, Pec, or their combinations would be considered as an interesting approach to improve postharvest quality characteristics of plum fruit.

## 1. Introduction

Fruits and vegetables are a great source of antioxidants, anthocyanins, phenolics, some vitamins and nutritional elements [1] which are associated with reduced risk of chronic health disorders [2,3]. Plums (*Prunus domestica* L.) are an important fruit, among the functional foods and nutraceuticals. Plums are a good source of antioxidants. They might help the human body to fight various diseases. However, plums have short postharvest life that results in loss of valuable and nutritional elements [4]. Plums quality rapidly declines after harvesting due to their high respiration rate. Consequently, after transportation and marketing process, they often do not reach consumers at their best status [5,6].

In recent years, applications of safer methods for fruit preservation are of a high significance. These safe methods usually have neither side effects on human and animal health, nor negative influences on the environment. Edible coatings are considered as one of the safe strategies. Edible coatings could improve fruit postharvest. So, application of edible coatings with natural origin such as proteins and polysaccharides has received a growing interest [7,8].

Polysaccharide-based edible coatings act as efficient oxygen blockers due to their well-arranged hydrogen bonded network structure but not as moisture barriers. The coatings are commonly colorless, oil-free and with low caloric content that often prolong the postharvest storability of fruit by reducing the dehydration and oxidative rancidity [7]. Moreover, polysaccharide-based edible coatings are highly stable, safe, nontoxic and biodegradable. Cellulose derivatives and pectin are two main groups of polysaccharide-based edible coatings [9].

Carboxymethylcellulose (CMC) is a cellulose derivative, anionic, linear, long-chain and high molecular weight compound [10,11]. CMC-based coatings mostly do not have odor, taste, and any toxic or allergic effects. They also are biodegradable, flexible, transparent, oil resistant, soluble-in-water and slightly permeable to oxygen, CO_2_, and moisture [12].

Pectin (Pec), main compound of plant cell walls, is a complex high molecular weight polysaccharide with branching structure [13,14], and an amorphous and colloidal carbohydrate [14]. Pec-based coatings are excellent barriers to O_2_ and CO_2_, in addition to their transparency, oil-resistance, and water solubility. They prevent moisture loss to some extent and eventually maintain the sensory aspects and quality of foods [14,15].

CMC-based edible coatings have been shown to be efficient in preserving postharvest quality of pear, papaya, mandarin and peach [16,17,18,19]. Pec-based ones preserved quality of peach, nectarine, fresh-cut apple, and persimmon [20,21,22,23]. Some studies reported the application of edible coatings on plum fruit, including chitosan [24], and carboxymethylcellulose, alone [8] or in combination with irradiation [6].

Given this background, few studies were performed using these polysaccharide-based edible coatings (CMC and Pec) on plum fruit during cold storage. CMC-based edible coatings (with the best effect at 1%) were effective in maintaining firmness and nutritional attributes (e.g., titratable acidity (TA), vitamin C (vit C), anthocyanin, flavonoid, antioxidant activity), decreasing polyphenol oxidase (PPO) and polygalacturonase (PG) and increasing peroxidase (POD) enzymes activities in plum during shelf life [8]. Moreover, no report of combination of the two coatings was observed on plum fruit. Accordingly, this study aims to investigate the influence of CMC- and Pec-based edible coatings, alone and combined, on some postharvest qualitative and enzymatic activities of plum in order to reduce postharvest losses of this fruit. Furthermore, the current survey might be a comprehensive evaluation of different qualitative characters especially antioxidant contents and enzymatic behavior of coated plum during cold storage.

## 2. Materials and Methods

### 2.1. Plant Materials

Fruits (*Prunus domestica* cv. “Golden Drop”) were obtained from an orchard in northwest of Iran (Shabestar) at their harvest stage (≈85 days after full bloom). They were ripe, firm, uniform in size and maturity and had an acceptable amount of TSS/TA with no damage or scar. The fruits, after washing with distilled water, were put on paper towels at room temperature to dry out and then coated with different polysaccharides.

### 2.2. Coating Treatments of Fruits

The experiment was done using three concentrations (0.5, 1 and 1.5%) of carboxymethylcellulose (CMC) and pectin (Pec) (Sigma Aldrich Chemic, Steinhein, Germany), both alone and in combination (total 16 including control and 15 treatments) (Table 1) in three replications and with sixty fruits for each replication.

Sampling was performed at weekly intervals for six weeks, 10 fruits per each sampling (30 fruits for each measurement). Coating treatment solutions (CMC and Pec) were obtained by dissolving them in purified water, while mixing at 60 °C, and glycerol 0.3% was added to plasticize the combination and then mixed again. Then, plum fruit were sunk in the homogenized solutions for 60 s and put at room temperature for one hour to dry. Subsequently, they were maintained at 4 °C and 85 ± 5% relative humidity on open plastic grids for six weeks. The control non-coated fruits were treated with purified water for 60 s. 

### 2.3. Evaluation of Fruit Quality 

#### 2.3.1. Measurement of Titratable Acidity (TA), Firmness, Vitamin C (vit C), Total Soluble Solids (TSS), pH, and Weight Loss

Titratable acidity was quantified through titration with 0.1 N NaOH up to pH 8.1. Firmness was assessed on both sides of peeled fruits utilizing 8-mm plunger of a manual penetrometer (Effegi, Milan, Italy). The vitamin C content of the fruit samples was verified by making use of a titrimetric method on the basis of the reduction of 2,6-dichlorophenolindophenol dye, as explained by AOAC [25]. A refractometer (PR-1; Atago Co., Ltd., Tokyo, Japan) was used to determine the TSS of the samples at 20 °C (expressed as %). A pH meter (Hanna Instruments, Milan, Italy) was used to record pH of the samples. Homogenized fruit samples (10 fruits) were used for measuring TSS, TA, and vit C. The results of TA and vit C were reported as g kg^−1^ on the basis of fresh weight. Percentage loss of initial weight was calculated as a unit for measuring weight loss. For each measurement, three technical replicates were included. 

#### 2.3.2. Total Phenolic Compounds, Total Anthocyanin, and Flavonoid Contents

Total phenolic compounds were determined using Folin–Ciocalteu reagent as reported by Singleton and Rossi [26]. Briefly, 1 g of the fleshy fruit was digested in 2 mL 1% HCl-methanol and centrifuged with a Universal 320R centrifuge (Andreas Heittich GmbH & Co., Tuttlingen, Germany) at 8000× *g* at 4 °C for 10 min. Then, the supernatant was utilized to measure the total phenolic compounds. To do this, 450 mL of distilled water and 2.5 mL of 10% Folin–Ciocalteu solution were added to 50 mL of extract and incubated in darkness. Then, the absorbance data were collected after 1.5 h incubation in darkness at 760 nm employing a Spekol 1500 spectrophotometer (Analytik Jena AG, Jena, Germany). The absorbance data were used to calculate the total phenolics and stated as g kg^−1^ gallic acid on the basis of fresh weight. A range of concentrations (50–500 μg mL^−1^) of gallic acid in 95% methanol was utilized as standard. The flesh and peel (1 g) of the fruit samples were properly sliced and used to extract anthocyanins in 2 mL of 1% HCl-methanol, and finally the extract was exploited to estimate the total anthocyanin content, as previously reported [27]. After centrifuging, the absorbance of the extract was quantified at 530 nm to calculate the total anthocyanin content as absorbance at 530 nm g^−1^ on a fresh weight basis. Total flavonoid content was determined according to the Woisky and Salatino method [28] with some modifications. Briefly, 1 g of peel and flesh of fruit samples were used to extract flavonoids in 4 mL 96% ethanol. After centrifuging, 700 μL 96% ethanol, 100 μL 10% aluminum chloride, 100 μL 1 M potassium acetate and at last 2.8 mL distilled water were added to 1300 μL of the extract (supernatant). Then, the absorbance of the solution was recorded at 415 nm after 30 min at room temperature. The findings were stated as g kg^−1^ quercetin on the basis of fresh weight. For standards, a range of quercetin concentrations (100–1000 μg mL^−1^) was used.

#### 2.3.3. Total Antioxidant Activity

The method, 1,1-Diphenyl-2-picryl-hydrazyl hydrate (DPPH), was used to verify the antioxidant activity. One gram of flesh and peel from fruit samples were chopped and extracted in 2 mL of 1% HCl-methanol and centrifuged subsequently [29]. Absorbance data were recorded at 517 nm after 15 min and the activity was analyzed and stated as percentage (%) using the formula:

% *Total antioxidant activity* = (*A_blank_* − *A_sample_*)/(*A_blank_*) × 100 (1)

#### 2.3.4. Peroxidase (POD), Polyphenol Oxidase (PPO) and Polygalacturonase (PG) Activities

One gram of flesh and peel from each fruit sample were smashed and extracted in 3 mL of 0.1 M phosphate buffer in an ice bath. After centrifugation, the supernatant was used as the crude enzyme extract. The activity of POD was evaluated according to the method explained by Arnnok et al. [30] with some modifications. The enzyme activity was assessed in 2 mL reaction mixture containing 0.1 M phosphate buffer, guaiacol, extract and H_2_O_2_. Guaiacol oxidation was examined by monitoring the increase in absorbance at 470 nm. The resulted data were stated as μmol tetraguaiacol min^−1^ g^−1^ on a fresh weight basis. The activity of PPO was calculated according to Jiang et al method [27] with modifications. A reaction mixture of 0.1 M phosphate buffer, 1 M 4-methylcatechol and enzyme solution was used to perform the PPO enzyme activity assay monitoring the upsurge in absorbance at 420 nm recorded for 90 s. The resulted data were expressed as μmol oxidized catechol min^−1^ g^−1^ on a fresh weight basis.

The activity of polygalacturonase enzyme was determined on the basis of the reducing groups released by PG and quantified by spectrophotometer [31]. One gram of flesh and peel from each fruit sample was smashed in 3 mL of 50 mM sodium acetate buffer and after centrifugation, 950 mL sodium acetate buffer and 1 mL 0.3% polygalacturonic acid were added to the supernatant (50 mL) and then the mixture was put at 30 °C for 45 min. Subsequently, the reaction mixture was stopped by boiling for 10 min after adding 800 mL 0.1 M borate buffer (pH 9.0) at 0 °C and 200 mL of 1% cyanoacetamide solution. After lowering the temperature, the absorbance at 276 nm was recorded. PG activity was noted as μmol D-galacturonic acid min^−1^ g^−1^ on the basis of fresh weight.

### 2.4. Statistical Analysis 

The study was carried out as a completely randomized design-based factorial experiment. After collecting data, subjects were assumed as a random factor and time as the repeated measure in a linear mixed model with Pec, CMC and time as fixed factors for a full factorial analysis of variance using the software IBM SPSS Statistics (version 21, SPSS Inc., Chicago, IL, USA). Marginal means were estimated based on maximum likelihood and least significant difference adjustment for multiple comparisons was done at *p* ≤ 0.05 and ultimately, these estimated marginal means were reported and their 95% confidence intervals were illustrated as error bars in the given graphs.

## 3. Results 

### 3.1. TA, Firmness, vit C, TSS, pH and Weight Loss

**Figure 1:**
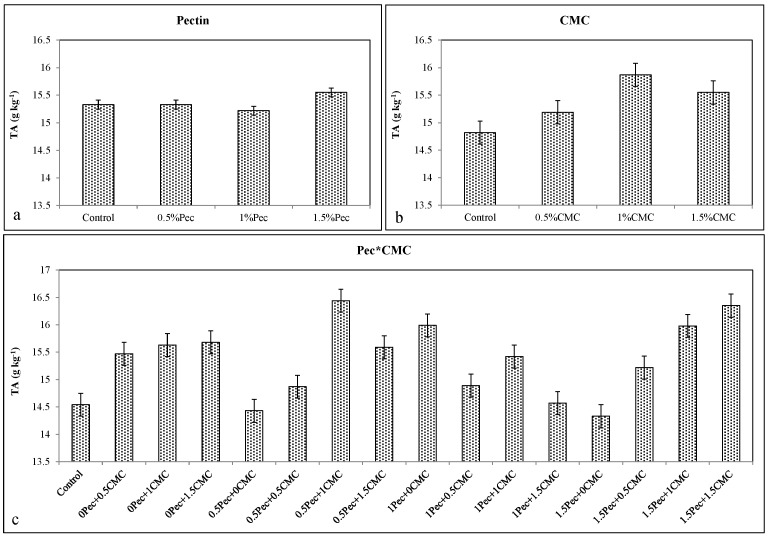
Effect of 0 (control), 0.5 (0.5% Pec), 1 (1% Pec) and 1.5 (1.5% Pec) % pectin-based edible coatings (**a**), 0 (control), 0.5 (0.5% CMC), 1 (1% CMC) and 1.5 (1.5% CMC) % carboxymethylcellulose-based edible coatings (**b**) and their combinations (**c**) on TA (titratable acidity) content during cold storage period. Data are the “estimated marginal means ± 95% confidence intervals”. The results were expressed on a fresh weight basis. The columns with non-overlapping error bars are significantly different (*p* ≤ 0.05).

Pec coating did not reduce TA content (Figure 1a), whereas CMC slightly increased TA values compared to the control (Figure 1b). All Pec and CMC combinations (except 1% Pec + 1.5% CMC ≈ 14.57 g kg^−1^) maintained TA contents of coated fruit with the high TA at 0.5% Pec + 1% CMC (≈16.44) and 1.5% Pec + 1.5% CMC (≈16.35 g kg^−1^) (Figure 1c).

**Figure 2:**
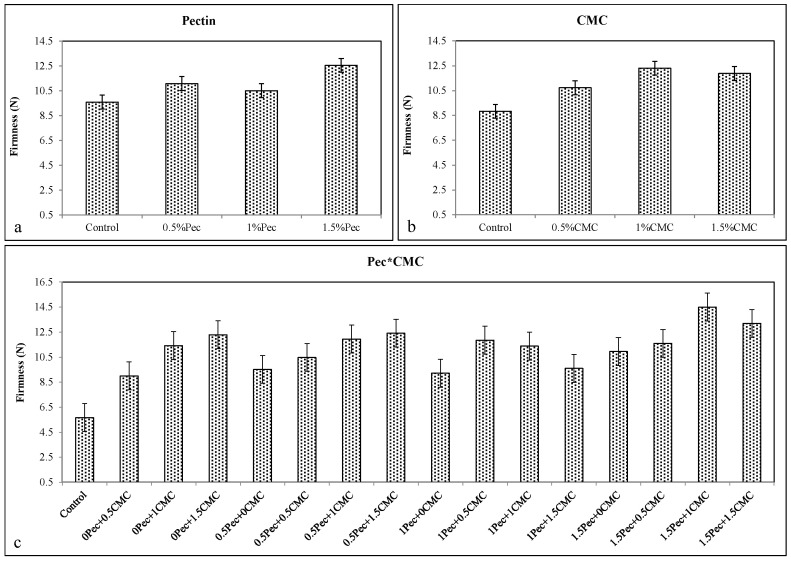
Effect of 0 (control), 0.5 (0.5% Pec), 1 (1% Pec) and 1.5 (1.5%Pec) % pectin-based edible coatings (**a**), 0 (control), 0.5 (0.5% CMC), 1 (1% CMC) and 1.5 (1.5% CMC) % carboxymethylcellulose-based edible coatings (**b**) and their combinations (**c**) on firmness during cold storage period. Data are the “estimated marginal means ± 95% confidence intervals”. The columns with non-overlapping error bars are significantly different (*p* ≤ 0.05).

Pec, CMC, and their combinations significantly increased the firmness of plum. In particular, the best results were achieved with 1.5% Pec (≈12.556 N) (Figure 2a), 1% CMC (≈12.31) (Figure 2b), and the combination 1.5% Pec + 1% CMC (≈14.495 N) (Figure 2c). 

**Figure 3:**
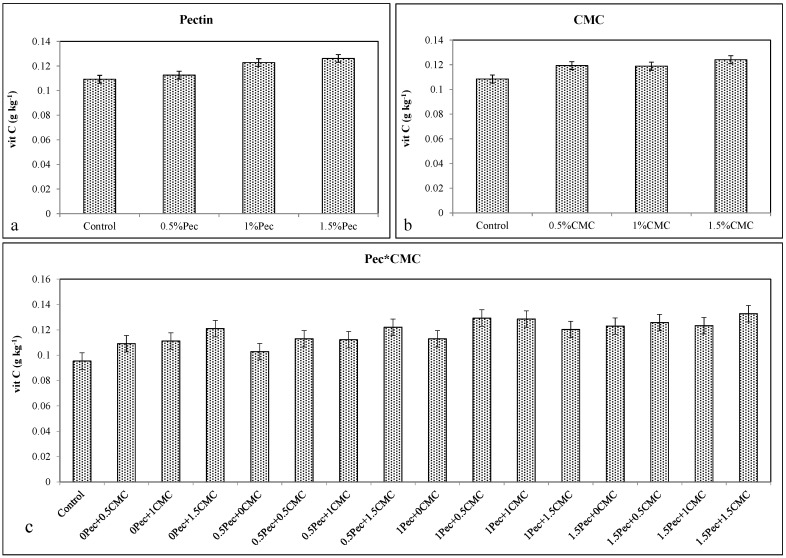
Effect of 0 (control), 0.5 (0.5% Pec), 1 (1% Pec) and 1.5 (1.5% Pec) % pectin-based edible coatings (**a**), 0 (control), 0.5 (0.5% CMC), 1 (1% CMC) and 1.5 (1.5% CMC) % carboxymethylcellulose-based edible coatings (**b**) and their combinations (**c**) on vitamin C (vit C) contents during cold storage period. Data are the “estimated marginal means ± 95% confidence intervals”. The results were expressed on a fresh weight basis. The columns with non-overlapping error bars are significantly different (*p* ≤ 0.05).

Pec coating at 1% and CMC at 0.5% significantly increased vit C concentration compared to control samples (Figure 3a,b). Among Pec*CMC combinations, 1.5% Pec + 1.5% CMC, 1% Pec + 0.5% CMC and 1% Pec + 1% CMC showed higher levels of vit C content (Figure 3c). The results suggest that coatings application, either alone or in combination, preserved vit C content during storage.

**Figure 4:**
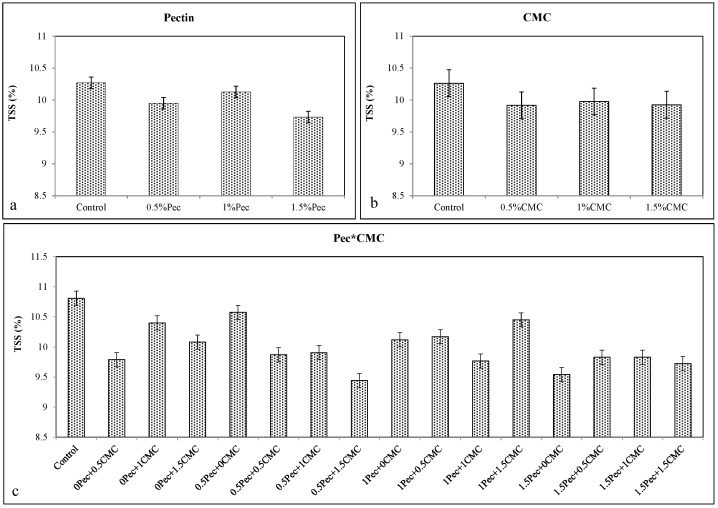
Effect of 0 (control), 0.5 (0.5% Pec), 1 (1% Pec) and 1.5 (1.5% Pec) % pectin-based edible coatings (**a**), 0 (control), 0.5 (0.5% CMC), 1 (1% CMC) and 1.5 (1.5% CMC) % carboxymethylcellulose-based edible coatings (**b**) and their combinations (**c**) on total soluble solids (TSS) contents during cold storage period. Data are the “estimated marginal means ± 95% confidence intervals”. The results were expressed on a fresh weight basis. The columns with non-overlapping error bars are significantly different (*p* ≤ 0.05).

The results for TSS values revealed that all Pec concentrations with the best result at 1.5% Pec (≈9.731%) (Figure 4a), all CMC concentrations (Figure 4b) and their combination at 0.5% Pec + 1.5% CMC (≈9.443%) (Figure 4c) significantly (*p* ≤ 0.01) reduced TSS of plum, as a positive result showing a lower breakdown in the fruit. 

**Figure 5:**
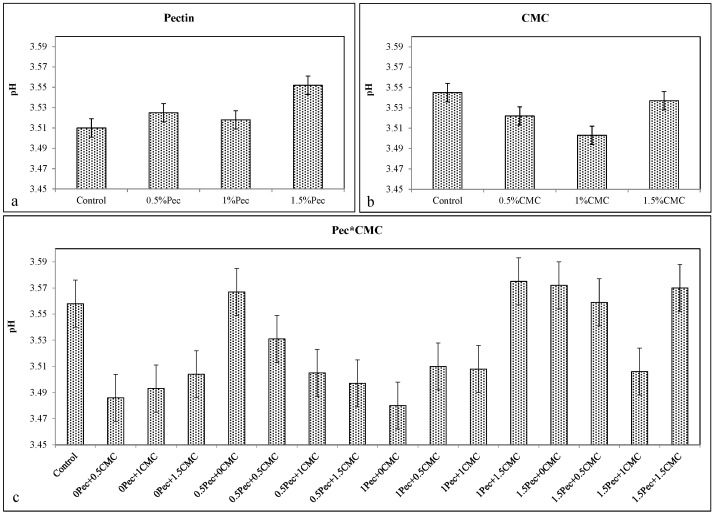
Effect of 0 (control), 0.5 (0.5% Pec), 1 (1% Pec) and 1.5 (1.5% Pec) % pectin-based edible coatings (**a**), 0 (control), 0.5 (0.5% CMC), 1 (1% CMC) and 1.5 (1.5% CMC) % carboxymethylcellulose-based edible coatings (**b**) and their combinations (**c**) on pH during cold storage period. Data are the “estimated marginal means ± 95% confidence intervals”. The columns with non-overlapping error bars are significantly different (*p* ≤ 0.05).

The pH values remained more or less stable in plum fruits coated with Pec (Figure 5a), CMC (Figure 5b) or their combinations (Figure 5c).

Weight loss of control fruits were around 15%. No considerable difference was detected between the control and coated fruit (CMC, Pec and Pec*CMC) in weight loss parameter (data not shown).

### 3.2. Total Phenolic Compounds, Total Anthocyanin and Flavonoid Contents and Total Antioxidant Activity

**Figure 6:**
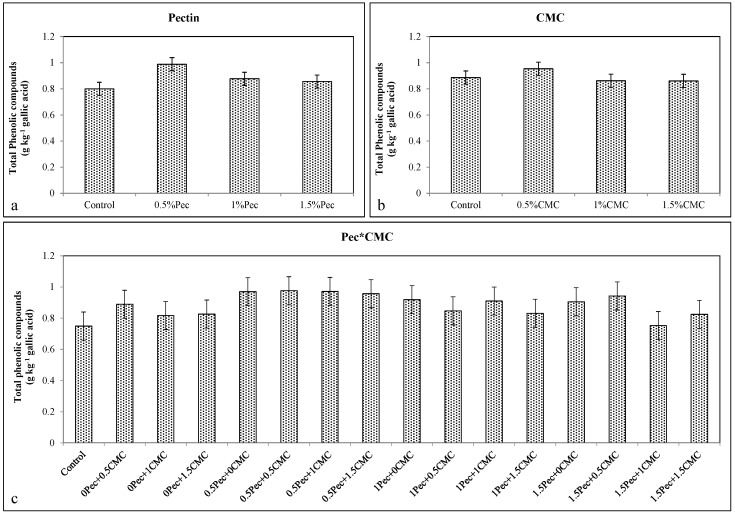
Effect of 0 (control), 0.5 (0.5% Pec), 1 (1% Pec) and 1.5 (1.5% Pec) % pectin-based edible coatings (**a**), 0 (control), 0.5 (0.5% CMC), 1 (1% CMC) and 1.5 (1.5% CMC) % carboxymethylcellulose-based edible coatings (**b**) and their combinations (**c**) on total phenolic compounds during cold storage period. Data are the “estimated marginal means ± 95% confidence intervals”. The results were expressed on a fresh weight basis. The columns with non-overlapping error bars are significantly different (*p* ≤ 0.05).

**Figure 7:**
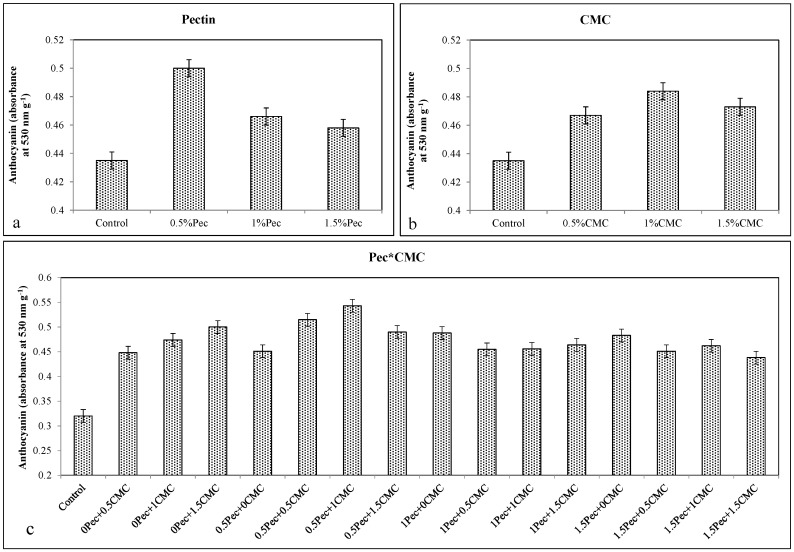
Effect of 0 (control), 0.5 (0.5% Pec), 1 (1% Pec) and 1.5 (1.5% Pec) % pectin-based edible coatings (**a**), 0 (control), 0.5 (0.5% CMC), 1 (1% CMC) and 1.5 (1.5% CMC) % carboxymethylcellulose-based edible coatings (**b**) and their combinations (**c**) on anthocyanin during cold storage period. Data are the “estimated marginal means ± 95% confidence intervals”. The results were expressed on a fresh weight basis. The columns with non-overlapping error bars are significantly different (*p* ≤ 0.05).

**Figure 8:**
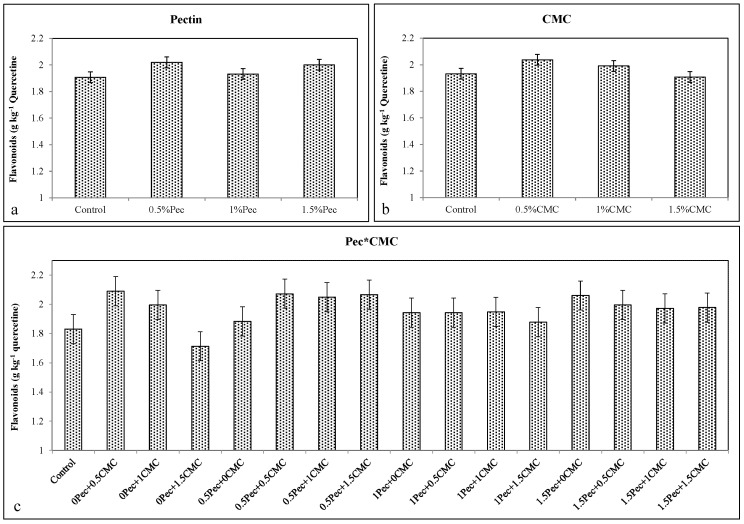
Effect of 0 (control), 0.5 (0.5% Pec), 1 (1% Pec) and 1.5 (1.5% Pec) % pectin-based edible coatings (**a**), 0 (control), 0.5 (0.5% CMC), 1 (1% CMC) and 1.5 (1.5% CMC) % carboxymethylcellulose-based edible coatings (**b**) and their combinations (**c**) on flavonoids contents during cold storage period. Data are the “estimated marginal means ± 95% confidence intervals”. The results were expressed on a fresh weight basis. The columns with non-overlapping error bars are significantly different (*p* ≤ 0.05).

**Figure 9:**
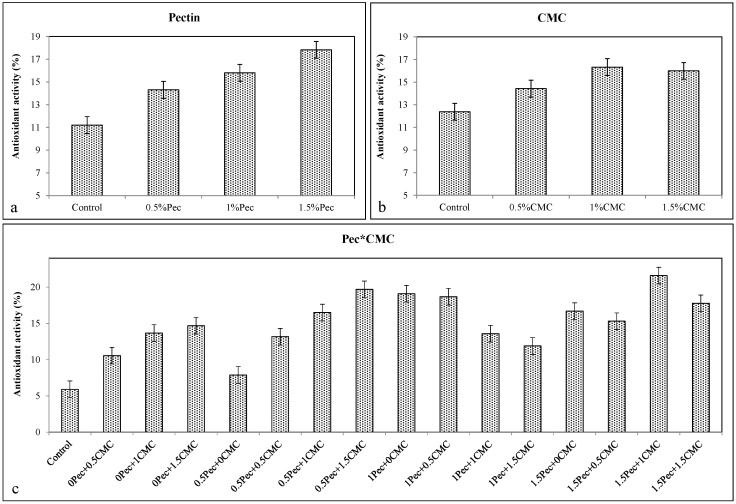
Effect of 0 (control), 0.5 (0.5% Pec), 1 (1% Pec) and 1.5 (1.5% Pec) % pectin-based edible coatings (**a**), 0 (control), 0.5 (0.5% CMC), 1 (1% CMC) and 1.5 (1.5% CMC) % carboxymethylcellulose-based edible coatings (**b**) and their combinations (**c**) on antioxidant activity based on the 1,1-Diphenyl-2-picryl-hydrazyl hydrate (DPPH) method during cold storage period. Data are the “estimated marginal means ± 95% confidence intervals”. The columns with non-overlapping error bars are significantly different (*p* ≤ 0.05).

The increase in total phenols, anthocyanins, and flavonoids after the coatings application (Pec or CMC alone or selected combinations) is in agreement with higher antioxidant activity detected in all coated fruits. Coating at 0.5% Pec increased total phenols (≈0.989 g kg^−1^ gallic acid) (Figure 6a), anthocyanins (≈0.5 A_530_ g^−1^) (Figure 7a), flavonoids (≈2.02 g kg^−1^ quercetin) (Figure 8a) and the antioxidant activity (≈14.31%) (Figure 9a). Likewise, the application of 0.5% CMC increased total phenols (≈0.954 g kg^−1^ gallic acid) (Figure 6b), anthocyanins (≈0.467 A_530_ g^−1^) (Figure 7b), flavonoids (≈2.04 g kg^−1^ quercetin) (Figure 8b) and the antioxidant activity (≈14.3%) (Figure 9b). Their combination (0.5% Pec + 0.5% CMC) resulted in higher total phenols (≈0.976 g kg^−1^ gallic acid) (Figure 6c), anthocyanins (≈0.515 A_530_ g^−1^) (Figure 7c), flavonoids (≈2.07 g kg^−1^ quercetin) (Figure 8c) and antioxidant activity (≈13.2%) (Figure 9c) than control samples. However, the highest values of total phenolic compounds (≈0.976 g kg^−1^ gallic acid) (Figure 6c), anthocyanins (≈0.543 A_530_ g^−1^) (Figure 7c), flavonoids (≈2.07 g kg^−1^ quercetin) (Figure 8c) and antioxidant activity (≈21.6%) (Figure 9c) were achieved by the combinations 0.5% Pec + 0.5% CMC, 0.5% Pec + 1% CMC, 0.5% Pec + 0.5% CMC and 1.5% Pec + 1% CMC, respectively.

### 3.3. POD, PPO and PG Enzymes Activities

**Figure 10:**
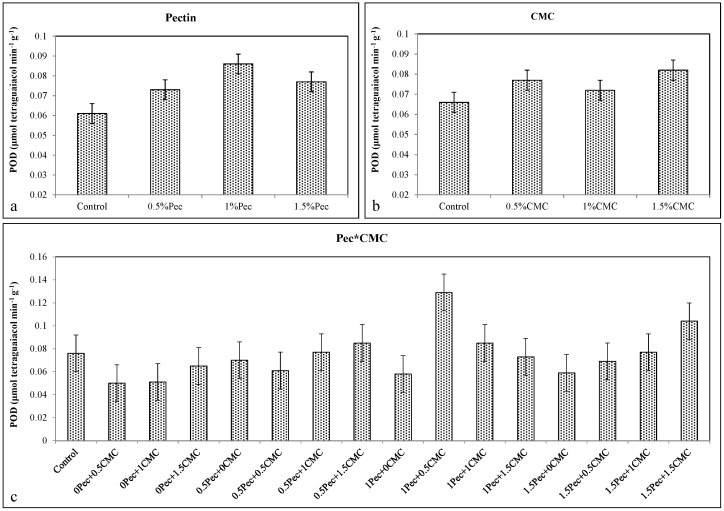
Effect of 0 (control), 0.5 (0.5% Pec), 1 (1% Pec) and 1.5 (1.5% Pec) % pectin-based edible coatings (**a**), 0 (control), 0.5 (0.5% CMC), 1 (1% CMC) and 1.5 (1.5% CMC) % carboxymethylcellulose-based edible coatings (**b**) and their combinations (**c**) on peroxidase (POD) enzyme activity during cold storage period. Data are the “estimated marginal means ± 95% confidence intervals”. The results were expressed on a fresh weight basis. The columns with non-overlapping error bars are significantly different (*p* ≤ 0.05).

Edible coatings determined higher POD activities compared to the control, with the maximum activity at 1% Pec concentration (≈0.086 μmol tetraguaiacol min^−1^ g^−1^) (Figure 10a) and 1.5% CMC concentration (≈0.082 μmol tetraguaiacol min^−1^ g^−1^) (Figure 10b). All combinations caused higher POD activities than the control (≈0.076) with the highest activities at 1% Pec + 0.5% CMC (≈0.129 μmol tetraguaiacol min^−1^ g^−1^) and 1.5% Pec + 1.5% CMC (≈0.104 μmol tetraguaiacol min^−1^ g^−1^) (Figure 10c).

**Figure 11:**
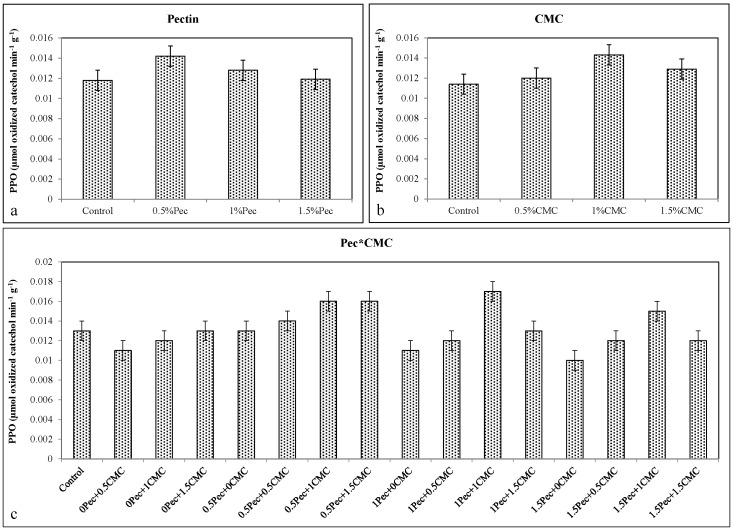
Effect of 0 (control), 0.5 (0.5% Pec), 1 (1% Pec) and 1.5 (1.5% Pec) % pectin-based edible coatings (**a**), 0 (control), 0.5 (0.5% CMC), 1 (1% CMC) and 1.5 (1.5% CMC) % carboxymethylcellulose-based edible coatings (**b**) and their combinations (**c**) on polyphenol oxidase (PPO) enzyme activity during cold storage period. Data are the “estimated marginal means ± 95% confidence intervals”. The results were expressed on a fresh weight basis. The columns with non-overlapping error bars are significantly different (*p* ≤ 0.05).

**Figure 12:**
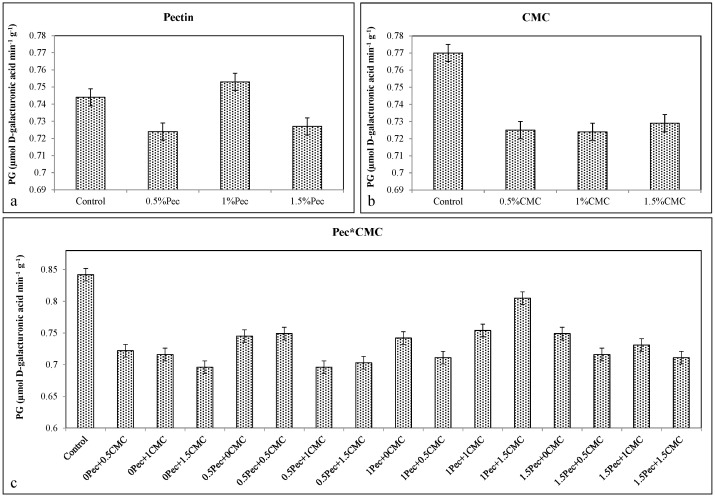
Effect of 0 (control), 0.5 (0.5% Pec), 1 (1% Pec) and 1.5 (1.5% Pec) % pectin-based edible coatings (**a**), 0 (control), 0.5 (0.5% CMC), 1 (1% CMC) and 1.5 (1.5% CMC) % carboxymethylcellulose-based edible coatings (**b**) and their combinations (**c**) on polygalacturonase (PG) enzyme activity during cold storage period. Data are the “estimated marginal means ± 95% confidence intervals”. The results were expressed on a fresh weight basis. The columns with non-overlapping error bars are significantly different (*p* ≤ 0.05).

Conversely, edible coatings significantly reduced PPO activity with the lowest values obtained with 1.5% Pec (≈0.014 μmol oxidized catechol min^−1^ g^−1^) (Figure 11a), 1% CMC (≈0.0137 μmol oxidized catechol min^−1^ g^−1^) (Figure 11b), and 1.5% Pec + 1.5% CMC (≈0.013 μmol oxidized catechol min^−1^ g^−1^) (Figure 11c). A similar effect was found for the PG activity. In this case, the lowest enzymatic activity was achieved after the application of 0.5% and 1.5% Pec (≈0.718 μmol D-galacturonic acid min^−1^ g^−1^) (Figure 12a), 0.5%–1.5% CMC (≈0.725 μmol D-galacturonic acid min^−1^ g^−1^) (Figure 12b), and 0.5% Pec + 1% CMC (≈0.696 μmol D-galacturonic acid min^−1^ g^−1^) (Figure 12c).

## 4. Discussion

The obtained results demonstrated 1% CMC, 1.5% Pec and 0.5% Pec + 1.5% CMC as the best treatments for the preservation of the nutritional value of plums during postharvest cold storage.

In general, degradation of organic acids into sugars through respiration process decreases TA during postharvest [16,32]. Moreover, utilization of organic acids as a carbon skeleton for synthesizing new compounds could be another reason for TA reduction [16]. Delay in fruit ripening [5] and maturation [33] caused by coating might reduce respiratory metabolisms involved in TA loss. Positive effects of coatings on TA maintenance have been previously reported [24,34]. The 1% CMC treatment could prevent TA loss of plum during shelf life [8]. However, in the current study, all CMC concentrations increased TA value almost in line with Panahirad et al. [8].

Decrease in cell wall enzymes activities might be a probable reason for firmness preservation as stated by Sanchis et al. [23] and Kumar et al. [24] due to ripening delay by coating application [6]. Polygalacturonase (PG) enzyme is one of the main softening enzymes in plum [35]. Reduction in PG activity after coating (Figure 12) also reflects importance of this enzyme in plum softening. PG activity relies on respiration and production of ethylene. Therefore, unavailability of O_2_ postpones biosynthesis of ethylene and later textural changes in coated fruit. Consequently, controlling O_2_ availability and modifying internal gas composition by edible coatings decrease oxidative metabolism and delay changes in the fruit texture [33,36]. This can be another possible reason for firmness preservation here observed. The existence of carboxylic groups in chemical structure of CMC may cause a positive effect on firmness preservation [18]. The reduction of the soluble pectin fractions might be another reason for this positive effect [6,16]. Positive effect of CMC- and Pec-based edible coatings on firmness preservation has been previously reported [8,34]. Martinez-Romero et al. [37] and Kumar et al. [38] also reported positive effects of rosehip oil added to Aloe vera gel and lac-based coatings on plum firmness, respectively.

Antioxidant activity of vit C causes its loss during postharvest storage [6,19]. Ascorbic-acid oxidase and polyphenol oxidase modify vit C content whose activities directly rely on O_2_ availability. The decrease in the respiration rate [39] as well as the modulation of the O_2_ and CO_2_ transmission rate through the coating layer [32] could explain the vitamin C preservation. Indeed, Oms-Oliu et al. [40] reported that the coatings based on pectin produced a reduction of O_2_ diffusion and an increase in CO_2_ in the coated pears. The reduction of the PPO activity (Figure 11) in the coated samples could also be related with the vitamin C preservation. Similar results were already reported [24,34,36]. The vit C results of the current study are in agreement with our previous studies [8,41].

TSS is an important quality parameter and its amount at harvest as well as during storage is important for consumer acceptance [35]. The thin layer of the coating on the fruit surface reduces evaporation, delays degradation and reduces respiration rate which, in turn, might have a positive effect on the prevention of TSS during storage. Positive effect of different edible coatings on TSS has been previously reported [24,32,34].

Some reports demonstrated that CMC- and Pec-based edible coatings could maintain a stable pH value [17,32,34] in agreement with our current findings.

Phenolic content contributes directly to the total antioxidant activity [3,42]. The upsurge in phenolic, anthocyanin and flavonoid contents was associated with a reduced PPO activity in the coated fruits as PPO triggers the oxidation of phenolics and flavonoids and degradation of anthocyanins [27,39]. In the current study, the reduction in PPO activity in the coated plums could be an explanation for the increase in phenolics, anthocyanins and flavonoids content. Pec-based edible coatings containing additives (such as anti-browning, apple fiber, and antioxidants) [20,40], as well as CMC [17] led to the accumulation of phenolic compounds. A positive effect of different coatings (alone or in combination with additives) on total phenolics has been reported [22,24,36,43]. Panahirad et al. [8] reported a negative effect of CMC on total phenolics, and a positive effect on anthocyanins and flavonoids contents. Pec-based edible coating enhanced phenolics, anthocyanins and flavonoids of plum and decreased PPO [41]. Chitosan coating caused higher quantities of phenolic compounds, anthocyanins and flavonoids [44] and a decline in the activity of PPO [27,45]. Other authors [20,22] reported higher flavonoid concentrations in the fruits treated with Pec–cinnamon leaf oil- and sodium alginate/Pec-based coatings, respectively.

The vit C has also antioxidative properties, however phenolic compounds along with anthocyanins and flavonoids are the main radical scavenging molecules [3,35,42]. The increase in the concentration of the mentioned compounds is in agreement with the enhancement of the total antioxidant activity and enzymatic activity of POD, a well-known antioxidant enzyme [27,39,46]. The enhancement of the total phenol content, anthocyanins, flavonoids, as well as the vit C preservation, the lower PPO activity, the higher POD activity, and the lower O2 permeability can all contribute, to different extents, to the higher antioxidant activity of the coated fruits. The increasing trend using the DPPH method in plum fruit during storage was previously reported [8,38,41,47]. The enhancement of antioxidant activity was also noticed in apple pieces coated with pectin in combination with pulse light treatment [48]. Oms-Oliu et al. [40] and Ayala-Zavala et al. [20] described similar positive effects using DPPH method on Pec-coated fruits. Guerriero et al. [22], Kumar et al. [24] and Ali et al. [49] all reported the preservation or the increase in the antioxidant activities of the fruits treated with sodium alginate/Pec-, chitosan- and gum Arabic-based coatings, respectively.

POD activity enhancement after application of Pec and CMC coatings has been reported by Ramirez et al. [21] and Panahirad et al. [8] in nectarine and plum, respectively. Panahirad et al. [41] additionally reported enhancement in POD activity on Pec-coated plums. Enhancement in POD activity could reduce the level of harmful radicals and consequently improves the antioxidant activity and postharvest quality of fruit maintaining their nutritional value. The positive effect of the applied coatings might be referred to formation of a semi-permeable barrier on fruit surface that restricts gas exchange and reduces water loss. This property delays physiological and biochemical changes that could result in quality preservation and strengthening of the antioxidant defense. In fact, slowing down the metabolic processes involved in senescence, ripening and decay might be considered as the main reason for POD enzyme enhancement by the applied coatings.

High PPO activity and phenolics oxidation are observed during storage, due to senescence-related processes, especially the destruction of biological barriers between PPO and polyphenols that activates the enzyme [19]. The application of coatings modulates the O_2_ exposure, decreasing the PPO activity [23,39]. In addition, reduction in pH can decrease the enzyme activity [23]. Decline in PPO activity by different coatings such as CMC, Pec, and chitosan has been previously reported [8,23,27,41,45]. Storage improvement by decreasing O_2_ availability, preservation of cellular compartmentation, protecting membrane structure from peroxidation and pH reduction due to slowing down of senescence and delaying of softening can be considered as possible reasons for the lower PPO activity.

## 5. Conclusions

The current survey reported positive effects of CMC- and Pec-based edible coatings, either alone or in combination with each other, on plum fruit through cold storage in terms of the measured parameters, except weight loss. The coatings especially improved vit C, total phenolics, anthocyanins and flavonoids contents and POD enzyme activity and decreased PPO and PG enzymes activities. CMC at 1% and Pec at 1.5% demonstrated the best results. Additionally, 0.5% Pec + 1.5% CMC is a good combined formulation for the preservation of the nutritional value of plum during postharvest. Thus, application of CMC and/or Pec and/or their combinations might be considered as a favorable and safe coating approach for extending and improving postharvest qualitative characteristics of plum fruit.

## Figures and Tables

**Table 1:** Treatment combinations of carboxymethylcellulose (CMC) (%) and pectin (Pec) (%) on fruits.

		CMC			
		0	0.5	1.0	1.5
**Pec**	0	1	2	3	4
	0.5	5	6	7	8
	1.0	9	10	11	12
	1.5	13	14	15	16
